# BDNF Differentially Affects Low- and High-Frequency Neurons in a Primary Nucleus of the Chicken Auditory Brainstem

**DOI:** 10.3390/biology13110877

**Published:** 2024-10-29

**Authors:** Kristine McLellan, Sima Sabbagh, Momoko Takahashi, Hui Hong, Yuan Wang, Jason Tait Sanchez

**Affiliations:** 1 Roxelyn and Richard Pepper Department of Communication Sciences and Disorders, Northwestern University, Evanston, IL 60208, USA; 2 Department of Neurobiology, Northwestern University, Evanston, IL 60208, USA; 3 Program in Neuroscience, Department of Biomedical Sciences, College of Medicine, Florida State University, Tallahassee, FL 32306, USA; 4 Feinberg School of Medicine, Northwestern University, Chicago, IL 60611, USA; 5 Oregon Hearing Research Center and Vollum Institute, Oregon Health & Science University, Portland, OR 97239, USA; 6 Knowles Hearing Research Center, Northwestern University, Evanston, IL 60208, USA

**Keywords:** neurotrophins, BDNF, development, potassium channels, sensory systems

## Abstract

Neurotrophins are proteins that mediate neuronal development using spatiotemporal signaling gradients. The chicken nucleus magnocellularis (NM), an analogous structure to the mammalian anteroventral cochlear nucleus, provides a model system in which signaling between the brain-derived neurotrophic factor (BDNF) and tyrosine receptor kinase B (TrkB) is temporally regulated. In the NM, TrkB expression is high early in development (embryonic [E] day 9) and is downregulated until maturity (E18–21). It is currently unknown how BDNF–TrkB signaling affects neuronal properties throughout development and across a spatial (i.e., frequency) axis. To investigate this, we exogenously applied BDNF onto NM neurons ex vivo and studied intrinsic properties using whole-cell patch clamp electrophysiology. Early in development (E13), when TrkB expression is detectable with immunohistochemistry, BDNF application slowed the firing of high-frequency NM neurons, resembling an immature phenotype. Current measurements and biophysical modeling revealed that this was mediated by a decreased conductance of the voltage-dependent potassium channels. Interestingly, this effect was seen only in high-frequency neurons and not in low-frequency neurons. BDNF–TrkB signaling induced minimal changes in late-developing NM neurons (E20–21) of high and low frequencies. Our results indicate that normal developmental downregulation of BDNF–TrkB signaling promotes neuronal maturation tonotopically in the auditory brainstem, encouraging the appropriate development of neuronal properties.

## Simple Summary

Neurotrophins mediate development in various sensory structures using spatiotemporal gradients. However, it is unclear how neurotrophins affect the development of the central auditory system and if they work in concert to establish a frequency (i.e., tonotopic) axis. We find that exogenous application of BDNF onto avian cochlear nucleus neurons causes significant changes to the intrinsic properties of high-frequency neurons but not of low-frequency neurons; additionally, this effect is seen only relatively early in development. Elucidating the impact of exogenous neurotrophins on the auditory brainstem is essential to understanding how neurotrophins establish spatial and temporal patterns within auditory nuclei. It also has vital consequences for neurotrophins as therapeutics in central auditory system-related disorders.

## 1. Introduction

Neurotrophins are signaling proteins that promote neuronal maturation in developing brain tissue and encourage synaptic plasticity in the adult brain [1]. The family of neurotrophins includes (but is not limited to) nerve growth factor (NGF), brain-derived neurotrophic factor (BDNF), and neurotrophin-3 (NT-3), all of which preferentially bind to various tyrosine receptor kinases (Trks) on the surface of nearby neurons [2,3]. This interaction begins a signaling cascade in developing brains that simultaneously promotes various developmental processes across neuronal and non-neuronal structures. These include cell survival [4,5,6], synapse strengthening [7,8,9], dendritic architecture shaping [10,11], neuropeptide synthesis [12], ion channel alterations [13,14], and glial processes like myelinogenesis [15] and microgliogenesis [16]. Specifically, neurotrophin signaling helps regulate neurons’ morphological and biophysical properties and promotes unique phenotypes critical for biologically relevant functions [17,18,19]. Characterizing the impact of neurotrophin signaling is essential for understanding neural circuit development and appropriately diagnosing and treating neurodevelopmental disorders stemming from altered developmental signaling [20,21].

One such circuit feature that is dependent on neurotrophin signaling is auditory tonotopy. In the peripheral auditory system, Trk receptors are expressed in spatial gradients that help establish tonotopic maps, or the spatial organization of sensory inputs across the perceptive range of high to low frequencies [17,22,23,24]. Tonotopic maps are a hallmark organizational pattern found in auditory structures, where neurons are arranged spatially to encode a wide range of auditory frequencies that each animal perceives. Previous studies have indicated the presence of neurotrophin receptors in the central auditory system, but they have not explicitly examined the role that neurotrophins play in development [25,26]. A recent report suggests that neurotrophin signaling maintains the tonotopic map in the mouse medial nucleus of the trapezoid body, an auditory structure critical for sound localization [27]. However, it is unknown whether similar tonotopic patterns of Trk receptors are present in other central auditory structures or how neurotrophins affect the ion channel properties of neurons across the tonotopic axis. The spatial and developmental effects of neurotrophins remain critically understudied, as their impact on the structure and function of central auditory nuclei are not fully elucidated.

The chicken embryo has been used for decades in auditory research because of the animal’s precocious hearing development and overlapping frequency range comparable to that of humans [19,28,29,30]. In the chicken nucleus magnocellularis (NM), an analogous brainstem structure to the mammalian anteroventral cochlear nucleus, neurotrophin receptors TrkB and TrkC are expressed [26], similar to the murine cochlea and spiral ganglion neurons [17,22,23]. In the chicken NM, TrkB (which binds BDNF) is highly expressed early in development (~embryonic [E] day 9) and is downregulated to negligible amounts later in development (E18–21). TrkC (which binds NT-3) is also expressed early in development but increases its expression until the embryo hatches (~E21). Despite this knowledge, it is unknown how neurotrophin signaling affects neuronal properties throughout development and in the NM’s spatial (i.e., tonotopic) gradient. This reveals critical gaps in our knowledge concerning the influence of neurotrophins on the development of a first-order central auditory structure. Neurons in the NM encode sound features with high temporal fidelity [31]. Yet, it is undetermined how neurotrophins may mediate the development of this unique phenotype essential for sound encoding. This study aims to address this knowledge gap.

Like other auditory structures, the NM exhibits tonotopic organization: low-frequency neurons are found caudolaterally, and high-frequency neurons are found rostromedially within the nucleus [32]. We previously reported that the exogenous application of NT-3 affects the intrinsic properties of low-frequency, but not high-frequency, NM neurons early (E13) and later (E18) in embryonic development [19]. This is likely due to a differential expression of TrkC or to intrinsic neuronal differences across the tonotopic axis. Here, we investigate how BDNF–TrkB signaling affects the development of neuronal intrinsic properties in the NM. Specifically, we tested whether exogenous BDNF application differentially affects neurons across the tonotopic axis (i.e., high vs. low frequency) or at different developmental time points (i.e., early versus later development). We conclude that BDNF application differentially affects the intrinsic properties of neurons across the tonotopic axis early in development: only high-frequency neurons demonstrate altered firing properties and a reduction in voltage-dependent potassium currents and channels. In contrast, low-frequency and late-developing neurons across all frequencies remain relatively unchanged by BDNF–TrkB signaling. These data are substantiated by immunohistochemistry, which suggests a higher expression of TrkB in high-frequency regions of the NM than in the low-frequency areas at the same early developmental stage. Our results reveal mechanisms underlying the role of neurotrophin signaling in establishing tonotopic neuronal properties within the central auditory system. Identifying the developmental processes for this specialized auditory structure is a crucial step in developing stem-cell or neurotrophin-based therapies for various developmental disorders affecting the central auditory system.

## 2. Materials and Methods

### 2.1. Animals

White leghorn chicken embryos (*Gallus gallus domesticus*) were used for the experiments outlined below. Embryonic sex was not determined in any experiment since embryonic sex determination is difficult and usually not attempted [29,30]. Experiments outlined in Section 2.4 and Section 2.5 were conducted on embryos at Northwestern University. Embryos used at Northwestern University were obtained from Michigan State University Poultry Teaching and Research Facility. All experiments followed Northwestern University Institutional Animal Care and Use Committee (IACUC) guidelines. Embryos were placed in Styrofoam incubators maintaining > 50% humidity and 100° Fahrenheit.

Experiments outlined in Section 2.2 and Section 2.3 were conducted on chicken embryos and mice (when noted) at Florida State University. Chicken embryos used at Florida State University were obtained from AVS Bio (Avian Vaccine Services, Norwich, CT, USA) and Michigan State University Poultry Teaching and Research Facility. Breeders for sighted FVB mice (FVB. 129P2-Pde6b+; #004828) were purchased from the Jackson Laboratory (Bar Harbor, ME, USA) and used to set up colonies in a Florida State University vivarium. All procedures were approved by the Florida State University Institutional Animal Care and Use Committees and conformed to National Institutes of Health guidelines.

Embryonic age was determined primarily based on incubation time (e.g., embryonic day 13 [E13]). In addition, we confirmed that E13 embryos qualitatively resembled the characteristics at Hamburger–Hamilton Stage 39, while embryos between E19 and 21 resembled Stage 45 [33].

### 2.2. TrkB Immunohistochemistry and Quantitative Microscopy

Brains were quickly dissected from chicken embryos at E13 (*n* = 4) and E19–20 (*n* = 4) [34,35]. Brainstems were isolated and fixed by submerging in 4% paraformaldehyde with 0.1 M phosphate buffer (PB) at 4 °C overnight. Brainstems were transferred to 30% sucrose in PB for at least three days. Coronal brainstem sections (50 μm thick for E13 and 40 μm thick for E19–20) were acquired using a freezing sliding microtome and collected in 0.01 M phosphate-buffered saline (PBS; pH of 7.4). Thicker sections were utilized for E13 animals since the brainstems are softer and more delicate to slice.

One-in-two (E13; 5–7 sections per animal) or one-in-four (E19–20; 6–8 sections per animal) series containing NM was used for TrkB immunohistochemistry. E13 sections were mounted on gelatin-coated slides, while E19–20 sections were handled free-floating. Sections were incubated overnight at 4 °C with a primary antibody solution containing anti-TrkB antibody (1:1000; Abcam Cat# ab18987, RRID: AB_444716, Waltham, MA, USA), anti-synaptotagmin 2 (Syt2; 1:5000; Developmental Studies Hybridoma Bank (DSHB) Cat# znp-1, RRID: AB_2315626), 0.3% Triton X-100, and 5% normal goat serum in PBS. After washing, sections were incubated overnight at 4 °C with Alexa Fluor secondary antibodies goat anti-rabbit 488 (1:1000; Thermo Fisher Scientific Cat# A32731, RRID: AB_2633280, Waltham, MA, USA), goat anti-mouse 568 (1:1000; Thermo Fisher Scientific Cat# A-11004, RRID: AB_2534072), and NeuroTrace 640/660 (1:1000; Thermo Fisher Scientific Cat# N21483, RRID: AB_2572212). After washing, sections were mounted on gelatin-coated slides (for E19–20) and coverslipped using Fluoromount-G mounting medium (Southern Biotech, Birmingham, AL, USA). No-primary control experiments were performed following the same procedure except without the primary antibody incubation.

Images were captured with an Olympus FV1000 confocal microscope (Center Valley, PA, USA). For illustration, image brightness, gamma, and contrast adjustments were performed in Adobe Photoshop (Adobe Systems, Mountain View, CA, USA). All adjustments were applied equally to all images from the same embryo. For quantifying the regional variation in TrkB immunoreactivity at E13, one section with the most considerable medial–lateral length of NM was selected from each embryo and imaged with a 20× lens. The mean TrkB intensity was measured by placing a square sample window of 80 µm in diameter in the medial and lateral portions of the NM using Fiji 2.14.0/1.54f software (National Institutes of Health, Bethesda, MD, USA). The mean was measured by encompassing the entire sample window or separately from the NeuroTrace-outlined somatic area and the remaining (neuropil) region. Additionally, the ratio of TrkB intensity to NeuroTrace intensity was measured. For statistical analyses, TrkB intensity was normalized to the medial NM of the same embryo. For quantifying somatic TrkB intensity, images were captured from the medial and lateral portions of the nucleus with a 60× oil-immersion lens. Images from the same embryo were captured with the same imaging parameters. Cells with an identifiable cytoplasmic boundary and a cross-sectional area of more than 80 µm^2^ based on NeuroTrace staining were selected for TrkB quantification. TrkB intensity was measured as the mean gray value of TrkB immunostaining of individual NM cells. For statistical analyses, TrkB intensity was normalized to the mean of all measured cells from the lateral NM of the same embryo and grouped from all cells and animals of the same NM region (medial or lateral). Normalization test (Shapiro–Wilk) and unpaired, two-tailed Student’s *t*-test with Welch’s correction were performed using GraphPad Prism 10.3.0 software (La Jolla, CA, USA). Data are presented as mean ± standard deviation in the figures, and *p* < 0.05 was statistically significant.

### 2.3. Slice Preparation for Electrophysiology

Acute brainstem slices were prepared from white leghorn chicken embryos. Embryos aged E13 and E20–21 were used in this study to measure intrinsic neuronal properties during developmental periods when TrkB expression is present and absent, respectively [26]. In addition, these ages represent developmental time points before and after hearing onset in chickens [28]. A maximum of 6 cells were collected per embryo to ensure that a minimum of 4 embryos were included in each dataset. As described previously [36,37], the brainstem was dissected and isolated in ice-cold, oxygenated low-Ca^2+^ and high-Mg^2+^ modified artificial cerebrospinal fluid (ACSF) containing the following (in mM): 130 NaCl, 2.5 KCl, 1.25 NaH_2_PO_4_, 26 NAHCO_3_, 3 MgCl_2_, 1 CaCl_2_, and 10 glucose. The ACSF was continuously bubbled with a 95% O_2_/5% CO_2_ mixture and maintained at a pH between 7.2 and 7.4 and osmolarity between 295 and 310 mOsm/L. Once isolated, the brainstem was affixed to the stage of a vibratome slicing chamber (Ted Pella, Inc., Redding, CA, USA) and submerged in the ACSF solution described above. Bilateral coronal slices were made (200 µm thick), and approximately 2–6 slices (depending on embryonic age) containing NM were taken across the tonotopic axis, from caudal (low-frequency) to rostral (high-frequency). High-frequency neurons were obtained from the most rostral slice of NM, confirmed by the medial location of NM within the slice and the presence of nucleus angularis. Low-frequency neurons were recorded from a caudal NM slice. The most caudolateral slices of NM, referred to as NMc, were not collected since NMc neurons have drastically different morphological and intrinsic properties compared with NM [38]. If NMc neurons were accidentally obtained, the experimenter discarded all neuronal recordings from that slice and began recording at the next more rostral slice to obtain low-frequency NM recordings.

Slices were collected in a custom holding chamber and allowed to equilibrate for 1 h at 22 degrees Celsius in normal ACSF containing the following (in mM): 130 NaCl, 2.5 KCl, 1.25 NaH_2_PO_4_, 26 NAHCO_3_, 1 MgCl_2_, 3 CaCl_2_, and 10 glucose. Normal ACSF was continuously bubbled with a 95% O_2_/5% CO_2_ mixture and maintained at a pH between 7.2 and 7.4 and osmolarity between 295 and 310 mOsm/L. Once slices were equilibrated, a target slice was transferred to a recording chamber mounted on an Olympus BX51W1 microscope (Center Valley, PA, USA) for electrophysiological experiments. The microscope was equipped with a 60× water immersion objective. The recording chamber was superfused continuously (Gilson Minipuls 3, Middleton, WI, USA) with normal ACSF at room temperature (20–22 °C, monitored by Warner Instruments, Hamden, CT, USA) at a rate of 1.5–2 mL/min.

### 2.4. Whole-Cell Patch Clamp Electrophysiology

Pipettes were pulled to a diameter of 1–2 µm and a resistance of 3–8 MΩ using a P-97 flaming/brown micropipette puller (Silicon Valley, CA, USA). An Axon Multiclamp 700B amplifier (Molecular Devices, Silicon Valley, CA, USA) was used for current and voltage clamp recordings. We filled the pipettes with an internal solution consisting of (in mM): 105 K-gluconate, 35 KCl, 1 MgCl_2_, 10 HEPES-K^+^, 5 EGTA, 4 4-ATP-Mg^2+^, and 0.3 4-Tris2GTP, with a pH adjusted between 7.3 and 4 with KOH. In voltage clamp, the liquid junction potential was −10 mV, and it is corrected for all data presented. Neuronal capacitance was corrected, and all neurons with a series resistance > 10 MΩ were discarded.

Neurons were perfused with ACSF only (i.e., control condition), ACSF combined with BDNF (100 ng/mL) [39], ACSF combined with BDNF and the TrkB antagonist ANA-12 (100 µM) [40], or ACSF combined with BDNF and the pan-Trk antagonist GNF 5837 (10 µM) [41]. We also conducted one experiment with NM neurons perfused with ACSF and ANA-12 without BDNF. Regardless of the condition, all slices were incubated in their respective ACSF for two hours before beginning electrophysiological recording, and the reagents were present in the bath during experiments.

All experiments were performed with synaptic blockers continuously perfused in the bath (100 µM picrotoxin, GABA_A_-receptor blocker; 20 µM CNQX, AMPA-receptor blocker; and 100 µM DL-APV, NMDA-receptor blocker). Pipettes were visually guided to NM, and the region was identified at 5× magnification by the tissue’s orientation and the neurons’ relative size. After a gigaohm seal was maintained on the neuronal membrane, patches were ruptured, and NM neurons were held at −70 mV until stable.

Neurons with resting membrane potentials below −50 mV for E13 embryos or −55 mV for >E20 embryos were included in the dataset. Datapoints outside two standard deviations from the mean were excluded as outliers. Input resistances were also recorded at various times to ensure that each patch was stable.

In voltage clamp experiments, neurons were held at voltages between −100 and +20 mV in increments of 5 mV for 150 ms. Steady-state potassium currents were quantified by taking the average current value across 1 ms at the end of the voltage command (e.g., 3 ms before the command ended). Since potassium currents were measured at the end of a long depolarizing step, they were not influenced by inward sodium or calcium currents, which are either transient or negligible in magnitude [42]. In current clamp experiments, we recorded action potential properties by injecting 100 ms of current ranging between −100 and 200 or more (if the neuron’s rheobase was higher) in 10 pA increments. The current threshold was defined as the current injection needed to produce an action potential 5 or more times out of 10 repetitions. Once the current threshold was obtained, neurons received ten repetitions of suprathreshold current injections (defined as currents 25% of their current threshold value) for 100 ms. The following active properties were averaged across ten traces. Action potential latency was determined as the time of the action potential peak amplitude. The rise rate was the maximum positive dV/dt during the action potential. The repolarization rate was the maximum negative dV/dt during the action potential. We recorded passive properties by injecting −10 pA of current for 100 ms. The time constant (T) was obtained by fitting a single exponential to the first 10 ms of the voltage response (for > E20 cells, the first 5 ms was used). The input resistance (R) was calculated by dividing the change in voltage (averaged over the last 5 ms of the current injection) by the current injection (−10 pA). The capacitance (C) was calculated using the equation C = T/R.

Recording protocols were run using Clampex acquisition and Clampfit 11.3 analysis software (Molecular Devices, Silicon Valley, CA, USA). Statistics and graphs were created using GraphPad Prism 10 (La Jolla, CA, USA). Unpaired *t*-tests with Welch’s corrections (for comparisons between two experimental groups) and ANOVA with Bonferroni multiple comparisons (for comparisons between three or more experimental groups) were used to test significance. All *p*-values < 0.05 were considered significant. In current clamp, individual dots within bar graphs represent data from one neuron. In voltage clamp, average data are displayed with standard error (SEM).

### 2.5. Biophysical Modeling

The Boltzmann curve was constructed by first separating the current values into two bins (−60, −25 and −20, 30). The cut-off value was selected by taking the value reported in the previous literature [36]. We then took the slope of the I-V curve at each midpoint of the voltage increment and then divided each value by the largest value to normalize the differential conductance (i.e., channel density),

(1) $$P_{O}=\frac{\frac{dI}{dV}}{G_{Max}}$$

to a probability ranging from 0 to 1.

The curve was then fitted with the Boltzmann curve [43],

(2) $$P_{O}=B+\frac{\left(1-B\right)}{1+e^{\left(\frac{V_{50}-V}{K}\right)}}$$

using nonlinear regression to extrapolate *V*_50_ (representing shift on open probability) and *K* (valence *z*, possibly indicative of the channel ionic environment or the stability of the intermediate states of the potassium channels) [43,44,45]. For the low-voltage gated channels, *B* was set to 0; *B* could not be set to 0 for the high-voltage gated channels due to the presence of the low-voltage current by the time the high-voltage gated channels opened.

To confirm the fit of *K* and *V*_50_ as well as determine whether *G_Max_* (maximal differential conductance, indicative of the maximum number of channels open) was statistically different between the control and the BDNF population for each developmental age and tonotopic region, the Boltzmann curve was then solved as a differential equation, where the equation was reversed to

(3) $$\frac{\frac{dI}{dV}}{G_{Max}}=B+(\frac{1-B}{1+e^{\frac{V_{50}-V}{K}}})$$

Since this is a separable first-order differential equation,

(4) $$\frac{dI}{dV}=\;B\cdot G_{Max}+G_{Max}\left(\frac{1\;-\;B}{1+e^{\frac{V_{50}\;-V}{K}}}\right)$$

the solution becomes

(5) $$I=B\cdot G_{Max}\;V+G_{Max}\;\left(1-B\right)\;Kln\left|e^{\frac{V}{K}}+e^{\frac{V_{50}}{K}}\right|+C$$

where *C* was obtained by using the Y-intercept of the I-V curve. The fit of *V*_50_ and *K* were then determined by performing nonlinear regression and determining the intervals for each parameter (*V*_50_, *K*, *G_Max_*). A one-way ANOVA was performed to see whether there was a difference for *G_Max_* between the control, BDNF-applied, and BDNF+ANA-12 applied populations. Post hoc Brown–Forsythe and Welch ANOVAs with multiple comparisons were completed to test significance at chosen voltage values (−42.5 mV for low-voltage activated potassium channel open probability and differential conductance, −2.5 mV for high-voltage activated potassium channel open probability, and +17.5 mV for high-voltage activated potassium channel differential conductance).

### 2.6. Reagents

BDNF was obtained from Abcam (#AB206642, Cambridge, UK), while ANA-12 (Cat. #4781) and GNF 5837 (Cat. #4559/10) were obtained from Tocris (Ellisville, MO, USA). All TrkB-related reagents were incubated with the neurons for two hours before recording, and they were continuously circulated within the bath during experiments.

## 3. Results

We first examined the distribution pattern of TrkB immunoreactivity in the developing NM to determine whether tonotopic differences were present. We then recorded from 189 neurons to assess the effects of TrkB activation by exogenous BDNF application on the intrinsic properties of NM neurons across spatial (i.e., tonotopic) and temporal (i.e., developmental) axes. Finally, we performed biophysical modeling on 47 recorded neurons to investigate the mechanisms of change in the ion channel properties. We studied two developmental time points: one early in development before the hearing onset in chicken embryos (E13) [28] and one late in development when the physiological properties of NM neurons are near auditory maturation (E20–21) [36].

### 3.1. TrkB Immunoreactivity in Developing NMs

The developmental profiles of TrkB and TrkC in NM neurons were previously characterized [26]. TrkB immunoreactivity in NM neurons was detected at early stages (E7–E11), while TrkC was present in NM neuronal bodies throughout development (E7–E20). We reexamined the distribution of TrkB immunoreactivity in NMs at E13 and E20. An anti-TrkB antibody that recognizes the c-terminus of the full-length mammalian TrkB was used. 

**Figure 1:**
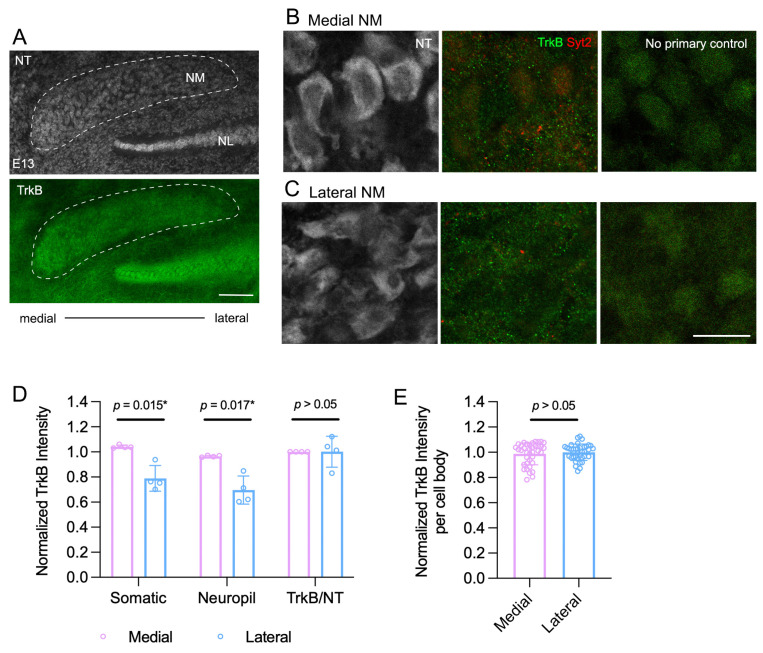
TrkB immunoreactivity in E13 NM neurons. (**A**) Low-magnification images of TrkB immunoreactivity and NeuroTrace (NT) counterstain. Note the gradient of overall TrkB intensity in NM with higher staining in the medial portion of the NM. Scale bar = 100 µm. NL, nucleus laminaris. (**B**,**C**) High-magnification images of TrkB signals from the medial (**B**) and lateral (**C**) NM. No-primary controls from each region are shown on the right. Note distinct anti-TrkB puncta, which were not present in the no-primary controls. Syt2 signal is low at this age. Scale bar = 20 µm. (**D**) Measurement of the mean intensity of TrkB over a sample window of 80 µm in diameter. “Somatic” TrkB was measured from anti-TrkB signals that overlap with NeuroTrace counterstain as a cell body marker. “Neuropil” TrkB was measured from anti-TrkB signals located outside of NeuroTrace-stained cell bodies. “TrkB/NT” was calculated by normalizing the somatic TrkB intensity to the NeuroTrace intensity within the sample window. Data were normalized to the total intensity of the medial NM of the same embryo. (**E**) Quantification of somatic TrkB intensity of individual neurons from the medium and lateral NM. TrkB intensity was measured from individual cell bodies as the mean gray value of TrkB immunostaining and normalized to the mean of all measured cells from the lateral NM of the same embryo. See Section 2 Materials and Methods for more details. Two-tailed Student’s *t*-tests with Welch’s correction were performed. * = *p* < 0.05.

In E13 chicken brainstems, the TrkB antibody displayed weak but above-background immunoreactivity in the NM (Figure 1). Bright anti-TrkB puncta were observed within and between neuronal cell bodies (Figure 1B,C). No obvious puncta were observed in sections with no-primary control, validating the specificity of this signal to antibody binding. The overall anti-TrkB signal was not uniform within the NM, often with the most robust staining found in the medial portion (Figure 1A). We thus compared the anti-TrkB intensity between the medial and lateral NM at both the population and individual neuron levels. The mean anti-TrkB intensity over a sample window of 80 µm in diameter was higher in the medial NM than in the lateral NM in somatic (*p* = 0.015) and neuropil (*p* = 0.017) regions (Figure 1D). However, there was no significant difference in the somatic anti-TrkB signal after normalization to NeuroTrace. Additionally, the somatic TrkB intensity measured from individual NM somata was comparable between the medial and lateral portions of the NM (Figure 1E; two-tailed unpaired *t*-test; *p* = 0.996; *n* = 38 neurons from medial NM and 37 neurons from lateral NM; see Section 4 Discussion). 

**Figure 2:**
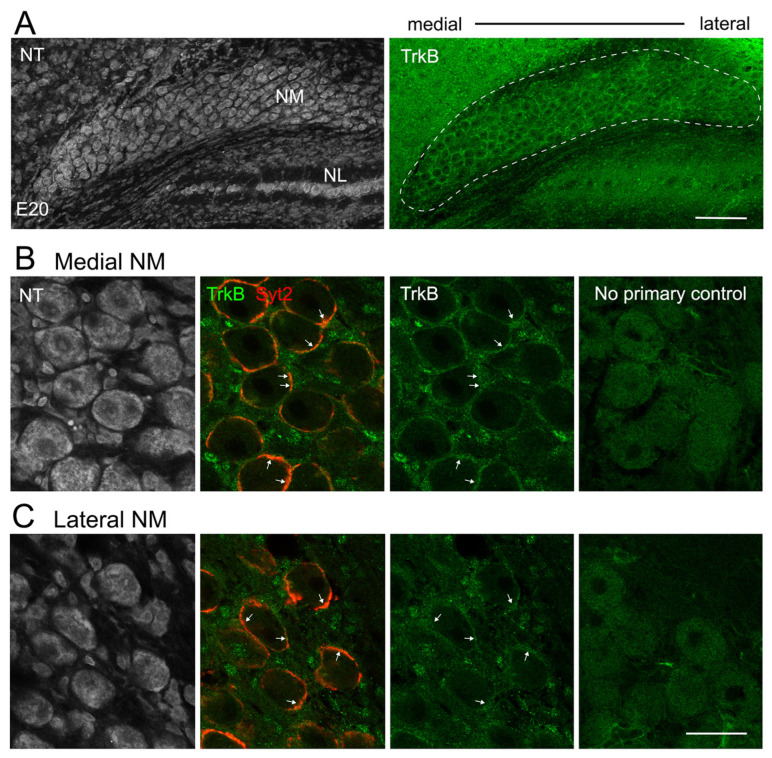
TrkB immunoreactivity in E20 NM neurons. (**A**) Low-magnification images of TrkB immunoreactivity and NeuroTrace (NT) counterstain. Scale bar = 100 µm. NL, nucleus laminaris. (**B**,**C**) High-magnification images of TrkB signals from medial (**B**) and lateral (**C**) NM. Arrows point out several TrkB-containing structures that overlap with Syt2-labeled presynaptic terminals. No-primary controls from each region are shown on the right. Scale bar = 25 µm.

Somatic staining of TrkB in NM cells disappeared at E19–20 (Figure 2A). Instead, TrkB immunoreactivity wrapped around NM cell bodies, suggesting auditory nerve presynaptic localization (Figure 2B,C). Double labeling of TrkB with Syt2, an auditory nerve endbulb terminal marker [46], confirmed this localization. This observation is consistent with the previous report that demonstrated no somatic NM TrkB expression at this stage of development [26], further confirming the specificity of the anti-TrkB antibody used. These observations support the presence of TrkB in NM neurons at E13. To examine whether the detected TrkB signals in E13 NM cells are functional, we next characterized neuronal responses to BDNF application. 

### 3.2. Electrophysiology in Early Embryonic Development (E13)

#### 3.2.1. High-Frequency NM Neurons in Early Embryonic Development

**Figure 3:**
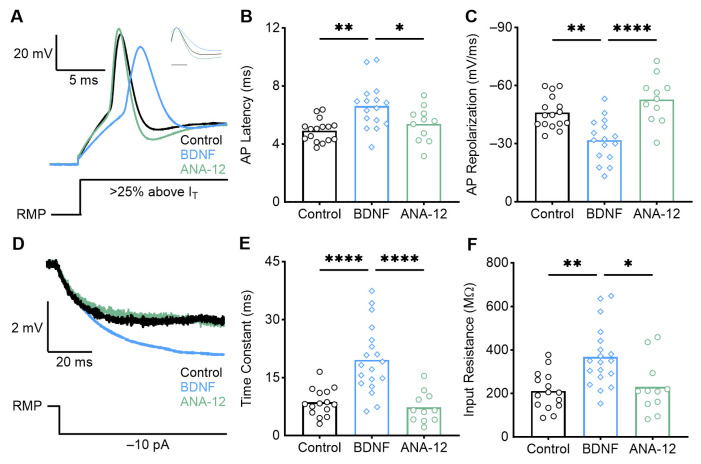
Early developing high-frequency neurons demonstrate altered active and passive properties after BDNF application. (**A**) Average representative voltage traces in response to a sustained current injection 25% above AP threshold (I_T_) for ACSF-only (control), BDNF, and ANA-12 (TrkB antagonist) conditions. Normalized peak amplitude AP shown in inset. Scale bar = 2 ms. A schematic of the current injection is shown below. BDNF application increased the AP latency (**B**) and slowed the AP repolarization rate (**C**) compared with control, and the effect is reversed with ANA-12. (**D**) Average representative voltage traces during a −10 pA current injection (schematic below). BDNF application increases the time constant (**E**) and input resistance (**F**) compared with control, and the effect is reversed with ANA-12. * = *p* < 0.05, ** = *p* < 0.01, and **** = *p* < 0.0001.

Slices were bathed in ACSF only (i.e., control, *n* = 16), ACSF with BDNF (*n* = 20), or ACSF with BDNF and the TrkB antagonist ANA-12 (*n* = 11). We also recorded from neurons bathed with ANA-12 without BDNF (*n* = 6) to confirm that ANA-12 alone minimally affects the NM intrinsic properties (Table A1). High-frequency neurons were found in the most rostromedial coronal slice of the NM. During current clamp recordings, BDNF application significantly affected the active intrinsic properties of high-frequency NM neurons (Figure 3). Following a 2 h bath application of BDNF, high-frequency neurons demonstrated significantly slower action potentials (APs) compared with control (ACSF only), quantified by an increase in AP peak latency (Figure 3B; *p* = 0.0012, control = 4.93 +/− 0.78 ms; BDNF = 6.62 +/− 1.59 ms). BDNF application also significantly slowed the AP repolarization rate (Figure 3C; *p* = 0.0010, control = −46.04 +/− 8.35 mV/ms; BDNF = −31.81 +/− 10.94 mV/ms). These changes were diminished when BDNF and the TrkB antagonist ANA-12 were applied concurrently, reverting to the control condition (latency: *p* = 0.038, ANA-12 = 5.38 +/− 1.212 ms; repolarization rate: *p* < 0.0001, ANA-12 = −52.82 +/− 11.95 mV/ms). There were no changes in the resting membrane potential, AP rise rate, AP current threshold, or AP peak amplitude between the control and BDNF conditions (Table A1). This suggests that BDNF interacted with TrkB to induce these intrinsic changes in high-frequency NM. BDNF–TrkB signaling slows the AP latency and repolarization rate of high-frequency NM neurons, causing them to resemble active properties from neurons at earlier developmental stages (<E13) [36].

High-frequency E13 NM neurons also exhibited an increase in passive membrane properties, including the time constant (Figure 3E, *p* < 0.0001, control = 8.62 +/− 3.55 ms; BDNF = 19.60 +/− 8.72 ms) and input resistance (Figure 3F, *p* = 0.0013, control = 211.00 +/− 86.25 MΩ; BDNF = 368.20 +/− 138.20 MΩ). These effects were reversed when both BDNF and ANA-12 were concurrently bath-applied (time constant: *p* < 0.0001, 7.28 +/− 3.99 ms; input resistance: *p* = 0.011, 230.19 +/− 123.02 MΩ). An increase in these passive membrane properties suggests that BDNF–TrkB signaling may cause changes in ion channel expression or alterations to NM morphology, the latter being previously reported [11].

**Figure 4:**
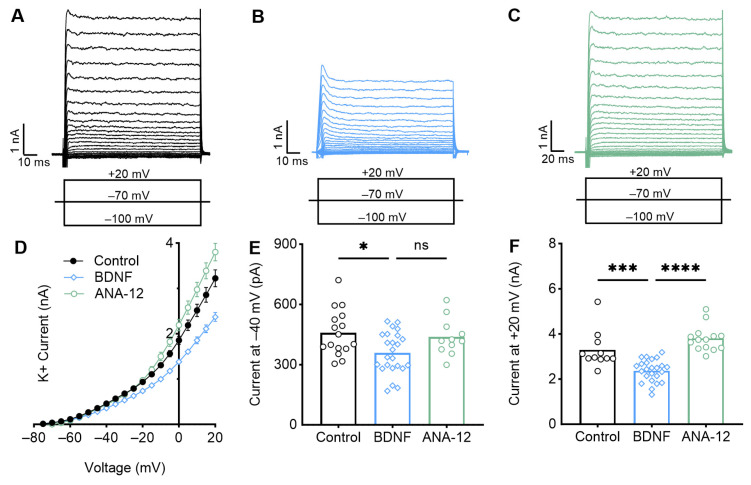
Early developing high-frequency neurons exhibit reduced potassium currents after BDNF application. Representative current traces for (**A**) control, (**B**) +BDNF, and (**C**) +ANA-12 neurons after voltage steps from −100 to +20 mV. Schematic voltage commands are shown below. Steady-state potassium currents were obtained 3 ms before the end of each voltage step and used to construct the I-V curve. (**D**) The I-V curve across all voltage commands demonstrates a significant reduction in potassium current across various low (**E**) and high (**F**) depolarizing voltage commands when BDNF is applied. This suggests a decrease in both low- and high-voltage activated potassium channels. Applying the TrkB antagonist ANA-12 fully reverses these effects at higher voltages but only partially reverses the effects at lower voltages. ns = *p* ≥ 0.05, * = *p* < 0.05, *** = *p* < 0.001, and **** = *p* < 0.0001.

Our voltage clamp recordings indicated that BDNF application caused a reduction in potassium currents across various voltage commands (Figure 4). BDNF application significantly reduced the magnitude of the outward steady-state potassium currents for low depolarizing voltages (Figure 4E, −40 mV: *p* = 0.014, control = 458.40 +/− 115.30 pA; BDNF = 358.84 +/− 102.83 pA) and high depolarizing voltages (Figure 4F, +20 mV: *p* = 0.0043, control = 3.10 + 0.88 nA; BDNF = 2.37 + 0.49 nA). When BDNF and ANA-12 were applied together, the resulting potassium currents partially reversed at lower voltages, albeit not statistically significantly (*p* = 0.11, 437.81 +/− 91.94 pA), and significantly reversed at higher voltages (*p* < 0.0001, 3.79 +/− 0.56 nA). This suggests that BDNF–TrkB reduced the overall magnitude of outward potassium currents in early developing high-frequency NM neurons, likely affecting both low- and high-voltage activated potassium channels and mediating the alteration of intrinsic properties.

Next, we conducted biophysical modeling to investigate how BDNF application may alter the expression and kinetics of steady-state potassium channels for E13 high-frequency NM neurons. We applied a Boltzmann curve to our E13 high-frequency voltage clamp data to model the potassium channels’ open probability and maximum differential conductance (i.e., the number of channels or channel density). We separated our results to investigate low-voltage activated (LVA; i.e., currents from holding voltages between −60 and −30 mV) and high-voltage activated (HVA; i.e., currents from holding voltages between −25 and +30 mV) potassium channels.

**Figure 5:**
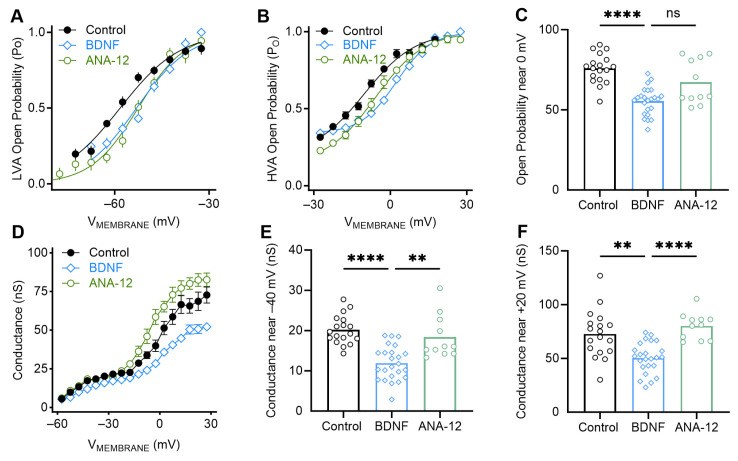
Biophysical modeling of E13 high-frequency neurons. (**A**) A sigmoidal curve derived from a Boltzmann distribution is fitted to the open probability of low-voltage activated potassium channels. (**B**) A sigmoidal curve derived from a Boltzmann distribution is fitted to the open probability of high-voltage activated potassium channels. (**C**) Open probability of high-voltage activated potassium channels at a high depolarizing voltage of −2.5 mV. BDNF significantly decreases the open probability of HVA potassium channels, which is partially reversed in the ANA-12 condition. (**D**) Differential conductance, analogous to potassium channel number, for low and high depolarizing voltages. Differential conductance for (**E**) a low depolarizing voltage of −42.5 mV and (**F**) a high depolarizing voltage of +17.5 mV demonstrate that BDNF decreases LVA and HVA potassium channel numbers. These are reversed with concurrent application of BDNF and ANA-12. ns = *p* ≥ 0.05, ** = *p* < 0.01, and **** = *p* < 0.0001.

The parameters calculated from the sigmoidal curves suggest that potassium channel open probability does not change with BDNF or ANA-12 application at low depolarizing voltages between −50 mV and −30 mV (Figure 5A, open probability at −42.5 mV: *p* = 0.43, control = 0.82 +/− 0.13; BDNF = 0.76 +/− 0.16; ANA-12 = 0.86 +/− 0.14). For potassium currents at high depolarizing voltages, the addition of BDNF significantly decreased the open probability compared with the control condition (Figure 5B,C; open probability at −2.5 mV: *p* < 0.0001, control = 0.55 +/− 0.91; BDNF = 0.38 +/− 0.73), and this was partially reversed with ANA-12 (*p* = 0.058, ANA-12 = 0.51 +/− 0.85). The change to open probability for high-voltage-dependent potassium channels suggests that BDNF application may alter the HVA potassium channel subtypes or alter the stability of the channels.

Differential conductance of low-voltage-dependent potassium channels, which may indicate channel number or function, significantly diminishes with BDNF application (Figure 5D,E; *p* < 0.0001, G_MAX_ at −42.5 mV: control = 20.19 +/− 3.60 nS; BDNF = 11.92 +/− 4.23 nS) and is reversed in the ANA-12 condition (*p* = 0.0091, ANA-12 = 18.39 +/− 5.47 nS). A similar trend is seen for high-voltage gated channels, with BDNF application significantly reducing the differential conductance compared with the control condition (Figure 5F, *p* = 0.0026; G_MAX_ at +17.5 mV: control = 72.76 +/− 22.09 nS; BDNF = 50.40 +/− 14.33 nS). This is again reversed in the ANA-12 condition (*p* < 0.0001, ANA-12 = 80.16 +/− 12.45 nS). These results suggest that BDNF decreases the channel conductance of low- and high-voltage activated potassium channels embedded in the neuronal membrane, mediating the reduction in steady-state potassium current magnitudes observed in our voltage clamp experiments.

Taken together, the application of BDNF onto E13 high-frequency NM causes biophysical alterations to the activity and subtype of both low- and high-voltage activated potassium channels, leading to an overall reduction in potassium current across various depolarizing voltages. This decrease in current causes significant changes in the active properties of these neurons, slowing the action potential waveforms and likely altering the neurons’ ability to encode auditory stimuli. Possible mechanisms for the changes in potassium channels are further elaborated in the Discussion.

#### 3.2.2. Low-Frequency NM Neurons in Early Embryonic Development

**Figure 6:**
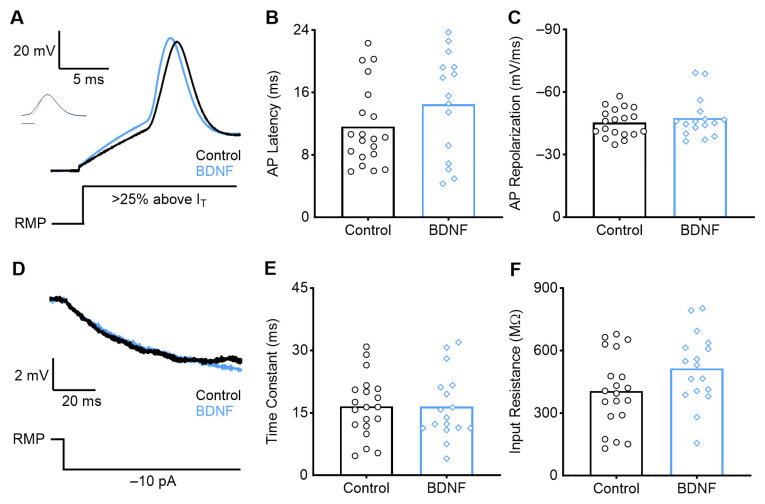
Early developing low-frequency neurons show no effect with BDNF application. (**A**) Average representative voltage traces in response to a sustained current injection 25% above AP threshold (IT) for control (ACSF only) and BDNF conditions. Normalized peak AP is shown in the inset (scale bar = 2 ms), and a schematic of the current injection is below. There is no difference in the AP latency (**B**) or AP repolarization rate (**C**) compared with control. (**D**) Average representative voltage traces during a −10 pA current injection (schematic below). BDNF application does not alter the time constant (**E**) or input resistance (**F**) compared with control. This demonstrates a tonotopic difference in the effect of BDNF relatively early in embryonic development.

Low-frequency NM neurons were identified in caudolateral coronal slices of the NM. Unlike high-frequency neurons at this developmental stage, BDNF did not significantly affect the active or passive properties of low-frequency E13 NM neurons (Figure 6). As described above, whole-cell current and voltage clamp electrophysiology experiments were performed on the control (*n* = 20) and BDNF (*n* = 17) conditions. Application of BDNF caused no significant differences in AP shape, latency (*p* = 0.18, control 11.64 +/− 5.17 ms; BDNF = 14.49 +/− 6.65 ms), or repolarization rate (*p* = 0.47, control= −45.38 +/− 6.69 mV/ms; BDNF = −47.51 +/− 9.74 mV/ms). There were no changes in the AP rise rate, current threshold, or peak amplitude between control and BDNF conditions (Table A1). There were also no differences in passive properties, including resting membrane potential, time constant, and input resistance. Our results parallel the differences in immunoreactivity we observed in the NM across the tonotopic axis. This suggests a differential expression of TrkB along the tonotopic axis at this earlier stage in development, where TrkB is more highly expressed in high-frequency NM neurons than in low-frequency neurons.

### 3.3. Electrophysiology in Late Embryonic Development (E20–21)

#### 3.3.1. High-Frequency NM Neurons in Late Embryonic Development

**Figure 7:**
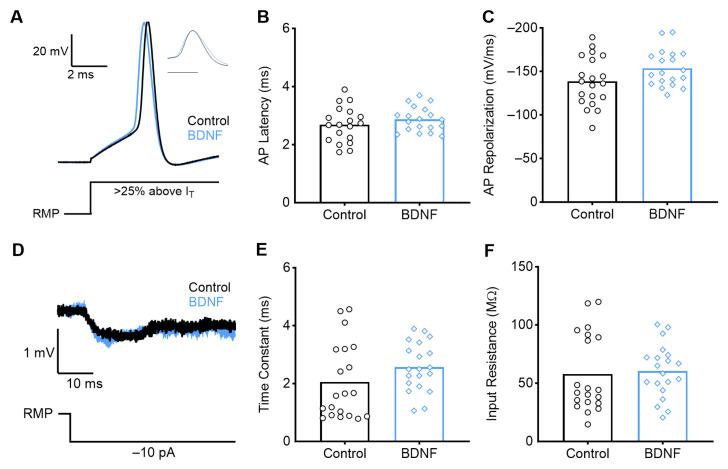
Late-developing high-frequency neurons show no effect with BDNF application. (**A**) Average representative voltage traces in response to a sustained current injection 25% above AP threshold (IT) for control and BDNF conditions. Normalized peak AP is shown in the inset (scale bar = 1 ms), and a schematic of the current injection is below. There is no difference in the AP latency (**B**) or AP repolarization rate (**C**) compared with control. (**D**) Average representative voltage traces during a −10 pA current injection (schematic below). BDNF application does not alter the time constant (**E**) or input resistance (**F**) compared with control. This is consistent with previous reports that suggest that late-developing NM neurons do not express the TrkB receptor.

At more mature embryonic stages (E20–21), NM neurons respond less drastically to BDNF application. Using whole-cell current and voltage clamp electrophysiology, we recorded from control (*n* = 20) and BDNF (*n* = 20) neurons. We found that late-developing high-frequency neurons exhibited no changes in active or passive membrane properties when bathed with BDNF (Figure 7). The E20–21 neurons were not different in any measure collected (Table A2).

This suggests no significant difference in the intrinsic properties or ion channel composition of high-frequency late-developing NM neurons in response to BDNF. This was expected, since our immunoreactivity demonstrated no somatic NM expression of TrkB at E20. Taken together, these results indicate that high-frequency NM neurons do not express the TrkB receptor at this late stage in development, as proposed by the previous literature [26], and thus, BDNF does not induce biophysical changes in passive and active membrane properties.

#### 3.3.2. Low-Frequency NM Neurons in Late Embryonic Development

**Figure 8:**
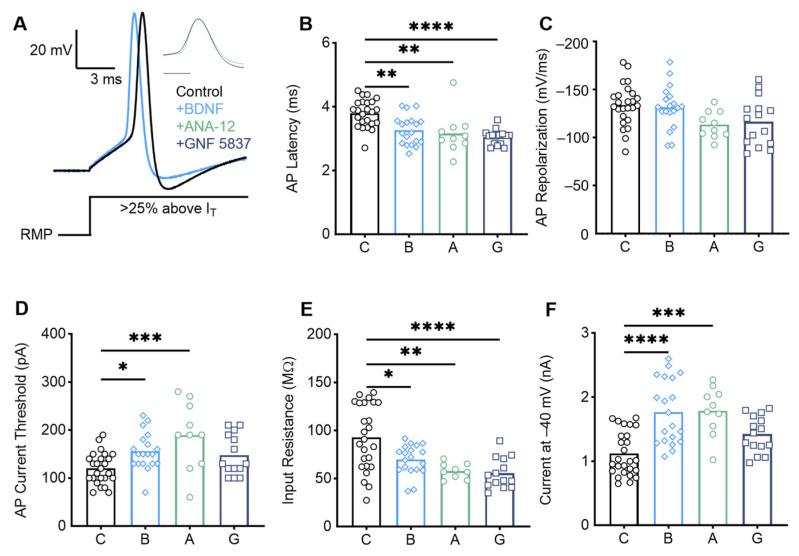
Late-developing low-frequency neurons demonstrate mixed intrinsic changes in response to BDNF. (**A**) Average representative voltage traces in response to a sustained current injection 25% above AP threshold (I_T_) for control (Control) and BDNF conditions. Normalized peak AP is shown in the inset (scale bar = 1 ms), and a schematic of the current injection is below. BDNF application decreases the AP latency (**B**) but does not change the AP repolarization rate (**C**) compared with control. These effects are not reversed with ANA-12 (TrkB antagonist) or GNF 5837 (pan-Trk receptor antagonist). The AP current threshold (**D**) is increased with BDNF application, and while this is not reversed in the ANA-12 condition, it is reversed in the GNF 5837 condition. BDNF application decreases the input resistance (**E**), which is not reversed with ANA-12 or GNF 5837. In voltage clamp, potassium currents significantly increase when held at −40 mV (**F**); this is not reversed when ANA-12 is added, but it is reversed in the GNF 5837 condition. C = Control condition, B = BDNF application, A = +ANA-12 application, G = +GNF 5837 application. * = *p* < 0.05, ** = *p* < 0.01, *** = *p* < 0.001, and **** = *p* < 0.0001.

E20 low-frequency NM neurons were bathed in either normal ACSF (*n* = 20), ACSF with BDNF (*n* = 20), ACSF with BDNF and the TrkB antagonist ANA-12 (*n* = 10), or ACSF with BDNF and the pan-Trk receptor antagonist GNF 5837 (*n* = 15). The results were consistent across low-frequency E20–21 NM neurons (Figure 8). BDNF application caused a decrease in AP latency (Figure 8B, *p* = 0.0014; control = 3.80 +/− 0.43 ms; BDNF = 3.27 +/− 0.46 ms) and an increase in AP current threshold (Figure 8C, I_T_; *p* = 0.047; control = 120.80 +/− 32.65 pA; BDNF = 155.80 +/− 38.49 pA). Surprisingly, when BDNF and the TrkB antagonist ANA-12 were applied concurrently, the effects were not reversed back to the control phenotype for AP latency (*p* = 0.94, 3.163 +/− 0.65 ms) or current threshold (*p* = 0.21, 189.0 +/− 69.83 pA). Since ANA-12 did not reverse the effects, we included a condition where BDNF was applied along with a pan-Trk antagonist, GNF 5837. Only in this condition were the effects for the current threshold reversed (control vs. GNF 5837 *p* = 0.25, GNF 5837 = 147.9 +/− 42.28 pA). The effects on latency, however, did not reverse (control vs. GNF 5837 *p* < 0.0001, GNF 5837 = 3.05 +/− 0.24 ms). There were no changes in the AP peak amplitude or repolarization rate (Table A2).

For passive properties, BDNF application did not significantly affect the membrane time constant (*p* = 0.53, control = 2.721 +/− 1.09 ms; BDNF = 2.36 +/− 0.69 ms), but it did significantly decrease the input resistance (Figure 8D, *p* = 0.01, control = 92.71 +/− 33.99 MΩ; BDNF = 69.61 +/− 15.51 MΩ). These effects were not reversed with either ANA-12 (*p* = 0.61, 57.78 +/− 7.97 MΩ) or GNF 5837 (control vs. GNF 5837 *p* < 0.0001, 55.49 +/− 15.89 MΩ). There were no changes in the resting membrane potential (Table A2).

The changes in excitability properties for low-frequency late-developing NM neurons were again revealed in voltage clamp recordings. BDNF application increased the magnitude of the outward steady-state potassium currents at low depolarizing voltages (Figure 8F, −40 mV, *p* < 0.0001, control = 1118.02 +/− 343.50 pA; BDNF = 1767.47 +/− 502.88 pA). The effect was not reversed in the presence of ANA-12 (*p* = 0.99, 1787 +/− 377.50 pA), but it was reversed when in the presence of the pan-Trk antagonist GNF 5837 (control vs. GNF 5837 *p* = 0.09, 1420 +/− 284.10 pA). This suggests that BDNF may increase the magnitude of low-voltage activated potassium currents late in development, but this is likely the result of interactions between BDNF and non-TrkB receptors (further discussed below).

## 4. Discussion

The differential effects of neurotrophin signaling across development and the tonotopic region mediate the development of distinct neuronal properties at a first-order central auditory brainstem nucleus. We show that early in development (i.e., before hearing onset), exogenous BDNF–TrkB signaling significantly alters the intrinsic properties of auditory neurons in the high-frequency region of the avian nucleus magnocellularis (NM)—an auditory brainstem structure analogous to the mammalian anteroventral cochlear nucleus. Interestingly, no effects were seen for low-frequency neurons at the same developmental stage. Later in development, when TrkB is negligible, BDNF–TrkB signaling has no effect; while some differences were seen in low-frequency neurons, these seem to be independent of BDNF–TrkB interactions. The impact of exogenous BDNF–TrkB signaling on high-frequency early-developing neurons is complementary to our previous report, which showed that exogenous NT-3-TrkC signaling alters the intrinsic properties of low-frequency NM neurons across development [19]. Thus, we propose that two different neurotrophin signaling pathways—with opposing spatiotemporal expressions—establish intrinsic neuronal differences along the tonotopic axis at a central auditory structure during normal development. This mirrors the tonotopic patterning of neurotrophin signaling (i.e., ligand–receptor interactions) previously reported using immunostaining in the murine auditory periphery [22] and central auditory brainstem [27].

### 4.1. Early Development

TrkB-mediated neuronal responses early in development (E13) are consistent with somatic TrkB immunoreactivity. A previous study failed to detect TrkB mRNAs and immunoreactivity in E13 NM neurons [26]. We observed weak but detectable TrkB immunoreactivity at this age using an antibody that recognizes the c-terminus of the protein, as confirmed by no-primary controls. At later developmental ages (E19–20), this antibody replicated the presynaptic localization of TrkB, as previously reported in chicken embryos [26]. Thus, the TrkB immunoreactivity we observed at E13 likely represents actual TrkB signals. 

Exogenous BDNF application onto high-frequency neurons early in development (E13) caused the NM neurons to resemble the phenotype of immature neurons (e.g., E11–12) [36]. Overall, the action potential was slowed, observed as a longer latency and a reduction in repolarization rate. There was also an increase in the membrane time constant and input resistance, suggesting that the neuron responded to current impulses slower than in the control condition. Since TrkB expression in the chicken embryo is high in early embryonic development (~E9), our results suggest that experimentally prolonging BDNF–TrkB signaling with high exogenous BDNF application prevents high-frequency neurons from developing into mature, adendritic neurons with precise action potential firing properties. This observation aligns with a previous study demonstrating that genetically prolonging TrkB expression in NM resulted in dramatic changes in neuronal morphology, including profuse dendrites for typically adendritic neurons and increased aberrant neuronal excitability [11]. However, mechanisms for the later result still needed to be identified.

Here, we fill this gap with ex vivo recordings that show that BDNF application caused a decrease in potassium currents at low and high depolarizing voltages, which mediates changes in intrinsic neuronal properties. Biophysical modeling further indicates that BDNF application onto high-frequency early-developing neurons lowered potassium channel conductance, suggesting that the number of membrane-bound low- (e.g., Kv1) and high- (e.g., Kv3) voltage-dependent potassium channels decreased. The mechanism of this decline could be via decreased potassium channel expression, endocytosis of channels from the membrane, or instability of the channels, causing a disruption in conductance. Regardless of the mechanism, this demonstrates a causal link between BDNF–TrkB signaling and the regulation of voltage-gated potassium channels, which are critical to establishing the precise functional phenotype of NM neurons [31]. While these mechanisms were identified using a high concentration of exogenous BDNF application, endogenous BDNF–TrkB signaling earlier in development likely affects potassium channels similarly, though to varying degrees. It should be noted that our biophysical model is an approximate representation of physiological mechanisms. We use it as a tool to speculate on channel mechanisms and as a guide for additional biological research. Quantifying the Kv subunit presence and gene expression can be an area of future investigation.

Alterations to potassium channels in response to BDNF application have been previously reported in other brain areas, including the hippocampus, olfactory bulb, and auditory system [17,39,47,48]. The interaction between BDNF and TrkB spurs an intracellular signaling cascade that promotes long-term potentiation and ion channel modulation within minutes [14,39,49,50]. It is likely that BDNF application during our experimental paradigm induced changes in potassium channels either directly, through channel phosphorylation and stability, or indirectly, by altering channel expression and kinetics [47].

While significant changes were reported for high-frequency neurons early in development, no changes were seen in low-frequency neurons at the same age. Our electrophysiology data demonstrates a robust differential expression in neurotrophin signaling between high-frequency and low-frequency regions. One limitation of this study is that although we observed a regional difference in the overall TrkB signal in NM, the current research does not clarify the cellular substrates underlying this phenomenon. The somatic TrkB signals were comparable between the medial and lateral regions, suggesting that the stronger TrkB immunofluorescence in the medial NM likely results from more closely packed NM neurons in this region. Additionally, the cellular location of TrkB outside of NM cell bodies at E13 remains unidentified. At this age, NM neurons possess many dendrites that are eventually pruned later in development [29]. Whether incoming axons contain TrkB at E13, like at E20, is also unknown. Thus, dendritic or axonal TrkB may contribute to the higher anti-TrkB signal in the medial NM. Indeed, TrkB puncta have been found on the surface and intracellularly in the dendrites and axons of mammalian neurons [51,52]. The TrkB immunoreactivity staining in the chicken brainstem does not offer the robust signals needed for unambiguous identification of the expression and localization of TrkB. This may be partially due to low levels of TrkB at this age or the lack of highly sensitive and specific antibodies for chicken TrkB. RNA analysis using RNAscope and genetic labeling of endogenetic chicken TrkB would offer the means to overcome these difficulties.

**Figure 9:**
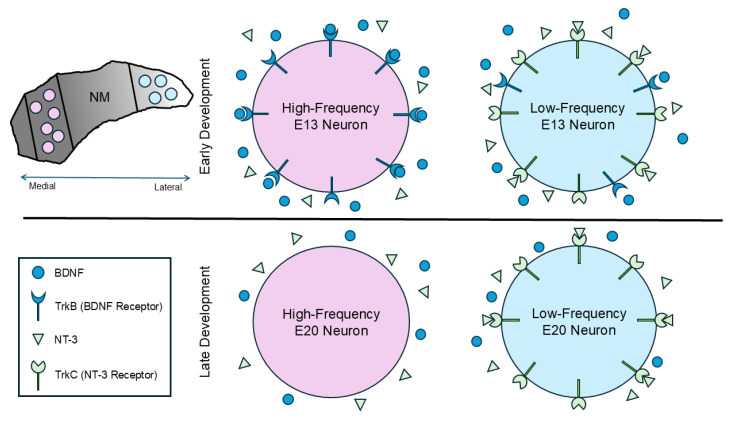
Neurotrophin signaling across tonotopy and development in the avian cochlear nucleus. NM (top left) is tonotopically organized, with high-frequency neurons found rostromedially and low-frequency neurons found caudolaterally (adopted from [31,53]). Early in development, high-frequency neurons exhibit some BDNF–TrkB signaling, while low-frequency neurons primarily exhibit NT-3–TrkC signaling. Later in development, before the chicken embryo hatches, high-frequency neurons do not express neurotrophin receptors, while low-frequency neurons still express TrkC [19,26].

The mammalian peripheral auditory system [17,22,23,25] and the murine superior olivary complex [27] have reported tonotopic gradients of neurotrophin signaling. This work and our previous report [19] introduce a parallel differential expression of neurotrophin signaling that supports the development of the tonotopic axis within the avian cochlear nucleus. This suggests that spatially distinct neurotrophin signaling may mediate the development of unique neuronal properties in high- versus low-frequency regions (Figure 9).

While our results implicate neurotrophins in establishing the intrinsic properties of NM neurons, these experiments were not observations of endogenous neurotrophin signaling across development. Instead, we used exogenous neurotrophin application ex vivo to represent how neurotrophin–receptor interactions may affect neurons in vivo. Our results and a previous study indicate that TrkB expression decreases across development in NM [26]. While normal endogenous BDNF levels across development are unknown, perhaps there is a parallel expression of BDNF and TrkB, as shown in other neural regions, including the auditory brainstem of the gerbil [54,55]. Our work also does not indicate the source of BDNF release for NM, which has been a topic of discussion for decades [11]. The most likely sources of BDNF are autocrine/paracrine interactions [56] implicated in regions across the auditory system [17,55]. A second possible source is from spiral ganglion neurons, as shown in the rat using RT-PCR [57]. More experiments are necessary to fill this knowledge gap.

### 4.2. Late Development

Few electrophysiological effects were seen for high- and low-frequency neurons at later, more mature stages of development (E20–21). Effects seen for low-frequency neurons indicate that they occur independently from BDNF–TrkB signaling. This confirms previous reports suggesting that TrkB is not expressed on NM neurons late in development [26]. We corroborated this with immunohistochemistry, which showed that TrkB is localized to auditory nerve presynaptic afferent terminals and not on the soma of NM neurons. While this indicates that BDNF–TrkB signaling may affect the presynaptic auditory nerve fibers, it does not affect neurons within NM at this developmental stage. Thus, in addition to a spatial difference, we have confirmed a temporal (e.g., developmental) difference in BDNF–TrkB signaling, which is detectable early in development but is no longer evident later in development.

Interestingly, we observed mixed effects of exogenous BDNF application on low-frequency E20–21 neurons. While some of these effects were reversed when applying the pan-Trk antagonist, GNF 5837, others were not. For the parameters that returned to control conditions after GNF 5837 application, we conclude that BDNF interacted with a different Trk receptor, possibly TrkC, to cause an increase in potassium currents and a quickening of active neuronal properties [58]. Since this effect is seen only in low-frequency neurons, this would align with previous findings suggesting that TrkC is more highly expressed in low-frequency neurons than in high-frequency neurons at this developmental stage [19,26]. It is unclear why applying BDNF and GNF 5837 concurrently did not cause a reversal of the action potential latency and input resistance back to the control condition. BDNF may weakly interact with other membrane receptors that subtly affect these intrinsic neuronal properties. In any case, BDNF did not seem to bind to TrkB to induce these changes.

A previous report found that 2 h of exogenous application of BDNF is sufficient to elicit electrophysiological changes [39]. While it is possible that increasing BDNF application time would induce more significant alterations in gene expression, the secretion of neurotrophins or other morphogens for less than 30 min is enough to observe changes in cellular morphology and intrinsic properties [59,60]. Interestingly, BDNF application produced distinct morphological changes in cultured rat hippocampal neurons in response to acute (~30 min) and sustained (8 h) exposure to BDNF [61]. Thus, while increasing incubation time may lead to more remarkable cellular changes, the time used in this report can effectively induce observed intrinsic and morphological changes.

It should be noted that in the ACSF-only conditions (i.e., control), the low-frequency neurons appeared less mature compared with the high-frequency neurons of the same age at both early (E13) and late (E20–21) embryonic stages. For example, the passive properties of E13 low-frequency neurons resemble a less mature (e.g., E11) phenotype compared with the E13 high-frequency neurons [36]. One possible explanation is that low-frequency NM neurons are consistently less mature than their high-frequency counterparts until embryonic maturity, as seen in NM’s downstream target, the nucleus laminaris [62]. These differences in the developmental timeline may cause a change in neurotrophin signaling. However, if the spatial differences in neuronal maturation caused the spatial difference in neurotrophin signaling, we would expect low-frequency neurons to have an even more substantial effect with BDNF application than high-frequency neurons since immature neurons express more TrkB [26]. Instead, we see the opposite effect, which suggests that the difference in neurotrophin signaling across the tonotopic axis depends on the spatial pattern of TrkB receptor expression across frequency and development.

In addition to the above points, future directions include using Western blot and quantitative PCR (qPCR) to evaluate potassium channel expression. Furthermore, variations in the quantity and subtypes of potassium channels can be tested using Kv1 and Kv3 immunostaining. Clinically, neurotrophins have been a potential treatment focus for various neurodegenerative diseases [63]. Understanding the effects of neurotrophin signaling on circuit mapping of the auditory brainstem provides evidence for how neurotrophins as therapeutics may affect the biophysical properties of the brain.

## 5. Conclusions

In sum, we report a spatiotemporal expression pattern of BDNF–TrkB signaling in the avian cochlear nucleus: BDNF influences the maturation of high-frequency neurons early in development, before the onset of hearing. We also demonstrate that neurotrophin signaling affects the intrinsic properties of auditory neurons by regulating the characteristics of voltage-dependent potassium channels that help to accurately encode auditory stimuli. This differential expression is spatially opposite to NT-3-TrkC signaling, which influences the maturation of low-frequency neurons across development. This suggests that two different neurotrophin signaling systems with opposing spatiotemporal expressions establish intrinsic neuronal differences along the tonotopic axis at a central auditory brainstem structure. Understanding the effects of exogenous neurotrophins on the development of the auditory system is an essential step in explaining how neurotrophin signaling mediates development in vivo. This research may also allow us to examine further the mechanisms and treatments for various central auditory channelopathies regulated by neurotrophin signaling.

## Data Availability

The data presented in this study are available upon request to the corresponding author.

## Appendix A

**Table A1:** Passive and active intrinsic properties for early-developing NM neurons, separated by frequency and experimental condition. HF = high frequency, LF = low frequency. Mean +/− standard deviation as well as *p*-value reported. RMP = resting membrane potential. AP = action potential. ** = *p* < 0.01 and **** = *p* < 0.0001.

Early Development (E13)	HF Control	HF +BDNF	HF +ANA-12	*p*-Value	LF Control	LF +BDNF	*p*-Value
RMP (mV)	−56.12 +/− 3.79	−57.77 +/− 3.94	−63.74 +/− 3.37	0.40	−60.11 +/− 2.92	−60.20 +/− 4.52	0.94
Time Constant (ms)	8.62 +/− 3.55	19.60 +/− 8.72	12.50 +/− 3.42	<0.0001 ****	16.57 +/− 7.30	16.47 +/− 7.99	0.96
Input Resistance (MΩ)	211.00 +/− 86.25	368.20 +/− 138.20	325.70 +/− 159.35	0.0013 **	405.60 +/− 177.46	514.12 +/− 172.19	0.068
AP Rise Rate (mV/ms)	83.23 +/− 16.17	75.45 +/− 22.43	84.76 +/− 15.84	0.52	63.74 +/− 16.22	62.84 +/− 19.54	0.88
AP Current Threshold (pA)	328.75 +/− 63.65	298.75 +/− 103.35	261.67 +/− 33.71	0.53	182.6 +/− 70.23	155.6 +/− 66.23	0.25
AP Peak Amplitude (mV)	72.64 +/− 6.54	76.94 +/− 9.03	73.23 +/− 5.07	0.28	66.61 +/− 9.15	68.65 +/− 9.45	0.52
AP Latency (ms)	4.93 +/− 0.78	6.62 +/− 1.59	5.36 +/− 0.63	0.0012 **	11.64 +/− 5.17	14.49 +/− 6.65	0.18
AP Repolarization Rate (mV/ms)	−46.04 +/− 8.35	−31.81 +/− 10.94	−56.51 +/− 6.18	0.0010 **	−45.38 +/− 6.69	−47.51 +/− 9.74	0.47

**Table A2:** Passive and active intrinsic properties for late-developing NM neurons, separated by frequency and experimental condition. HF = high frequency, LF = low frequency. Mean +/− standard deviation as well as *p*-values are reported. RMP = resting membrane potential. AP = action potential. * = *p* < 0.05 and ** = *p* < 0.01.

Late Development (E20–21)	HF Control	HF +BDNF	*p*-Value	LF Control	LF +BDNF	*p*-Value
RMP (mV)	−66.04 +/− 3.71	−67.64 +/− 2.30	0.11	−62.97 +/− 1.66	−63.32 +/− 3.56	0.34
Time Constant (ms)	2.06 +/− 1.31	2.57 +/− 0.84	0.15	2.72 +/− 1.09	2.36 +/− 0.69	0.53
Input Resistance (MΩ)	57.89 +/− 33.37	60.40 +/− 22.83	0.78	92.71 +/− 33.99	69.61 +/− 15.51	0.01 *
AP Rise Rate (mV/ms)	206.52 +/− 53.40	222.37 +/− 44.62	0.078	220.60 +/− 27.74	208.40 +/− 47.99	0.73
AP Current Threshold (pA)	333.90 +/− 120.10	308.00 +/− 81.86	0.45	120.80 +/− 32.65	155.80 +/− 38.49	0.047 *
AP Peak Amplitude (mV)	89.55 +/− 12.06	88.68 +/− 8.72	0.13	86.58 +/− 7.42	82.76 +/− 10.28	0.51
AP Latency (ms)	2.69 +/− 0.61	2.87 +/− 0.43	0.29	3.80 +/− 0.43	3.27 +/− 0.46	0.0014 **
AP Repolarization Rate (mV/ms)	−138.60 +/− 28.07	−153.50 +/− 20.96	0.067	−134.0 +/− 21.51	−131.70 +/− 22.06	0.98

## References

[1] Reichardt L.F. (2006). Neurotrophin-regulated signalling pathways. Philos. Trans. R Soc. Lond. B Biol. Sci..

[2] Bothwell M. (1995). Functional interactions of neurotrophins and neurotrophin receptors. Annu. Rev. Neurosci..

[3] Barbacid M. (1994). The Trk family of neurotrophin receptors. J. Neurobiol..

[4] Levi-Montalcini R., Angeletti P.U. (1963). Essential role of the nerve growth factor in the survival and maintenance of dissociated sensory and sympathetic embryonic nerve cells in vitro. Dev. Biol..

[5] Johnson E.C., Guo Y., Cepurna W.O., Morrison J.C. (2009). Neurotrophin roles in retinal ganglion cell survival: Lessons from rat glaucoma models. Exp. Eye Res..

[6] Gillespie L.N., Zanin M.P., Shepherd R.K. (2015). Cell-based neurotrophin treatment supports long-term auditory neuron survival in the deaf guinea pig. J. Control. Release.

[7] Schinder A.F., Poo M. (2000). The neurotrophin hypothesis for synaptic plasticity. Trends Neurosci..

[8] Wan G., Gomez-Casati M.E., Gigliello A.R., Liberman M.C., Corfas G. (2014). Neurotrophin-3 regulates ribbon synapse density in the cochlea and induces synapse regeneration after acoustic trauma. eLife.

[9] Ji L., Borges B.C., Martel D.T., Wu C., Liberman M.C., Shore S.E., Corfas G. (2024). From hidden hearing loss to supranormal auditory processing by neurotrophin 3-mediated modulation of inner hair cell synapse density. PLoS Biol..

[10] McAllister A.K., Lo D.C., Katz L.C. (1995). Neurotrophins regulate dendritic growth in developing visual cortex. Neuron.

[11] Schecterson L.C., Sanchez J.T., Rubel E.W., Bothwell M. (2012). TrkB downregulation is required for dendrite retraction in developing neurons of chicken nucleus magnocellularis. J. Neurosci..

[12] Nawa H., Pelleymounter M.A., Carnahan J. (1994). Intraventricular administration of BDNF increases neuropeptide expression in newborn rat brain. J. Neurosci..

[13] Lesser S.S., Sherwood N.T., Lo D.C. (1997). Neurotrophins differentially regulate voltage-gated ion channels. Mol. Cell. Neurosci..

[14] Rose C.R., Blum R., Kafitz K.W., Kovalchuk Y., Konnerth A. (2004). From modulator to mediator: Rapid effects of BDNF on ion channels. Bioessays.

[15] Dermitzakis I., Manthou M.E., Meditskou S., Miliaras D., Kesidou E., Boziki M., Petratos S., Grigoriadis N., Theotokis P. (2022). Developmental Cues and Molecular Drivers in Myelinogenesis: Revisiting Early Life to Re-Evaluate the Integrity of CNS Myelin. Curr. Issues Mol. Biol..

[16] Dermitzakis I., Manthou M.E., Meditskou S., Tremblay M.E., Petratos S., Zoupi L., Boziki M., Kesidou E., Simeonidou C., Theotokis P. (2023). Origin and Emergence of Microglia in the CNS-An Interesting (Hi)story of an Eccentric Cell. Curr. Issues Mol. Biol..

[17] Adamson C.L., Reid M.A., Davis R.L. (2002). Opposite actions of brain-derived neurotrophic factor and neurotrophin-3 on firing features and ion channel composition of murine spiral ganglion neurons. J. Neurosci..

[18] Desai N.S., Rutherford L.C., Turrigiano G.G. (1999). BDNF regulates the intrinsic excitability of cortical neurons. Learn. Mem..

[19] Takahashi M., Sanchez J.T. (2020). Effects of Neurotrophin-3 on Intrinsic Neuronal Properties at a Central Auditory Structure. Neurosci. Insights.

[20] Hefti F. (1994). Neurotrophic factor therapy for nervous system degenerative diseases. J. Neurobiol..

[21] Gillespie L.N., Shepherd R.K. (2005). Clinical application of neurotrophic factors: The potential for primary auditory neuron protection. Eur. J. Neurosci..

[22] Schimmang T., Tan J., Muller M., Zimmermann U., Rohbock K., Kopschall I., Limberger A., Minichiello L., Knipper M. (2003). Lack of Bdnf and TrkB signalling in the postnatal cochlea leads to a spatial reshaping of innervation along the tonotopic axis and hearing loss. Development.

[23] Fritzsch B., Pirvola U., Ylikoski J. (1999). Making and breaking the innervation of the ear: Neurotrophic support during ear development and its clinical implications. Cell Tissue Res..

[24] Green S.H., Bailey E., Wang Q., Davis R.L. (2012). The Trk A, B, C’s of neurotrophins in the cochlea. Anat. Rec..

[25] Hafidi A., Moore T., Sanes D.H. (1996). Regional distribution of neurotrophin receptors in the developing auditory brainstem. J. Comp. Neurol..

[26] Cochran S.L., Stone J.S., Bermingham-McDonogh O., Akers S.R., Lefcort F., Rubel E.W. (1999). Ontogenetic expression of trk neurotrophin receptors in the chick auditory system. J. Comp. Neurol..

[27] Wollet M., Kim J.H. (2022). Brain-Derived Neurotrophic Factor Is Involved in Activity-Dependent Tonotopic Refinement of MNTB Neurons. Front. Neural Circuits.

[28] Jones T.A., Jones S.M., Paggett K.C. (2006). Emergence of hearing in the chicken embryo. J. Neurophysiol..

[29] Jhaveri S., Morest D.K. (1982). Neuronal architecture in nucleus magnocellularis of the chicken auditory system with observations on nucleus laminaris: A light and electron microscope study. Neuroscience.

[30] Yamada R., Kuba H. (2021). Dendritic synapse geometry optimizes binaural computation in a sound localization circuit. Sci. Adv..

[31] Hong H., Sanchez J.T. (2018). Need for Speed and Precision: Structural and Functional Specialization in the Cochlear Nucleus of the Avian Auditory System. J. Exp. Neurosci..

[32] Rubel E.W., Parks T.N. (1975). Organization and development of brain stem auditory nuclei of the chicken: Tonotopic organization of n. magnocellularis and n. laminaris. J. Comp. Neurol..

[33] Hamburger V., Hamilton H.L. (1951). A series of normal stages in the development of the chick embryo. J. Morphol..

[34] Wang X., Zorio D.A.R., Schecterson L., Lu Y., Wang Y. (2018). Postsynaptic FMRP Regulates Synaptogenesis In Vivo in the Developing Cochlear Nucleus. J. Neurosci..

[35] Wang X., Kohl A., Yu X., Zorio D.A.R., Klar A., Sela-Donenfeld D., Wang Y. (2020). Temporal-specific roles of fragile X mental retardation protein in the development of the hindbrain auditory circuit. Development.

[36] Hong H., Rollman L., Feinstein B., Sanchez J.T. (2016). Developmental Profile of Ion Channel Specializations in the Avian Nucleus Magnocellularis. Front. Cell. Neurosci..

[37] Wang X., Hong H., Brown D.H., Sanchez J.T., Wang Y. (2017). Distinct Neural Properties in the Low-Frequency Region of the Chicken Cochlear Nucleus Magnocellularis. eNeuro.

[38] Hong H., Wang X., Lu T., Zorio D.A.R., Wang Y., Sanchez J.T. (2018). Diverse Intrinsic Properties Shape Functional Phenotype of Low-Frequency Neurons in the Auditory Brainstem. Front. Cell. Neurosci..

[39] Youssoufian M., Walmsley B. (2007). Brain-derived neurotrophic factor modulates cell excitability in the mouse medial nucleus of the trapezoid body. Eur. J. Neurosci..

[40] Montalbano A., Baj G., Papadia D., Tongiorgi E., Sciancalepore M. (2013). Blockade of BDNF signaling turns chemically-induced long-term potentiation into long-term depression. Hippocampus.

[41] Jeon M.T., Moon G.J., Kim S., Choi M., Oh Y.S., Kim D.W., Kim H.J., Lee K.J., Choe Y., Ha C.M. (2020). Neurotrophic interactions between neurons and astrocytes following AAV1-Rheb(S16H) transduction in the hippocampus in vivo. Br. J. Pharmacol..

[42] Rathouz M., Trussell L. (1998). Characterization of outward currents in neurons of the avian nucleus magnocellularis. J. Neurophysiol..

[43] Hodgkin A.L., Huxley A.F. (1952). A quantitative description of membrane current and its application to conduction and excitation in nerve. J. Physiol..

[44] Chowdhury S., Chanda B. (2012). Estimating the voltage-dependent free energy change of ion channels using the median voltage for activation. J. Gen. Physiol..

[45] Guidelli R., Becucci L., Aloisi G. (2020). Role of the time dependence of Boltzmann open probability in voltage-gated proton channels. Bioelectrochemistry.

[46] MacLeod K.M., Pandya S. (2022). Expression and Neurotransmitter Association of the Synaptic Calcium Sensor Synaptotagmin in the Avian Auditory Brain Stem. J. Assoc. Res. Otolaryngol..

[47] Jonas E.A., Kaczmarek L.K. (1996). Regulation of potassium channels by protein kinases. Curr. Opin. Neurobiol..

[48] Tucker K., Fadool D.A. (2002). Neurotrophin modulation of voltage-gated potassium channels in rat through TrkB receptors is time and sensory experience dependent. J. Physiol..

[49] Minichiello L., Calella A.M., Medina D.L., Bonhoeffer T., Klein R., Korte M. (2002). Mechanism of TrkB-mediated hippocampal long-term potentiation. Neuron.

[50] Nieto-Gonzalez J.L., Jensen K. (2013). BDNF Depresses Excitability of Parvalbumin-Positive Interneurons through an M-Like Current in Rat Dentate Gyrus. PLoS ONE.

[51] Drake C.T., Milner T.A., Patterson S.L. (1999). Ultrastructural localization of full-length trkB immunoreactivity in rat hippocampus suggests multiple roles in modulating activity-dependent synaptic plasticity. J. Neurosci..

[52] Gomes R.A., Hampton C., El-Sabeawy F., Sabo S.L., McAllister A.K. (2006). The dynamic distribution of TrkB receptors before, during, and after synapse formation between cortical neurons. J. Neurosci..

[53] Kuba H., Ohmori H. (2009). Roles of axonal sodium channels in precise auditory time coding at nucleus magnocellularis of the chick. J. Physiol..

[54] Ernfors P., Merlio J.P., Persson H. (1992). Cells Expressing mRNA for Neurotrophins and their Receptors During Embryonic Rat Development. Eur. J. Neurosci..

[55] Tierney T.S., Doubell T.P., Xia G., Moore D.R. (2001). Development of brain-derived neurotrophic factor and neurotrophin-3 immunoreactivity in the lower auditory brainstem of the postnatal gerbil. Eur. J. Neurosci..

[56] Schecterson L.C., Bothwell M. (1992). Novel roles for neurotrophins are suggested by BDNF and NT-3 mRNA expression in developing neurons. Neuron.

[57] Zha X.M., Bishop J.F., Hansen M.R., Victoria L., Abbas P.J., Mouradian M.M., Green S.H. (2001). BDNF synthesis in spiral ganglion neurons is constitutive and CREB-dependent. Hear. Res..

[58] Philo J., Talvenheimo J., Wen J., Rosenfeld R., Welcher A., Arakawa T. (1994). Interactions of neurotrophin-3 (NT-3), brain-derived neurotrophic factor (BDNF), and the NT-3.BDNF heterodimer with the extracellular domains of the TrkB and TrkC receptors. J. Biol. Chem..

[59] Park H., Poo M.M. (2013). Neurotrophin regulation of neural circuit development and function. Nat. Rev. Neurosci..

[60] McQuate A., Latorre-Esteves E., Barria A. (2017). A Wnt/Calcium Signaling Cascade Regulates Neuronal Excitability and Trafficking of NMDARs. Cell Rep..

[61] Ji Y., Lu Y., Yang F., Shen W., Tang T.T., Feng L., Duan S., Lu B. (2010). Acute and gradual increases in BDNF concentration elicit distinct signaling and functions in neurons. Nat. Neurosci..

[62] Sanchez J.T., Wang Y., Rubel E.W., Barria A. (2010). Development of glutamatergic synaptic transmission in binaural auditory neurons. J. Neurophysiol..

[63] Meldolesi J. (2017). Neurotrophin receptors in the pathogenesis, diagnosis and therapy of neurodegenerative diseases. Pharmacol. Res..

